# Supplementary material for: A Critical View of the Application of the APEX Software (Aqueous Photochemistry of Environmentally-Occurring Xenobiotics) to Predict Photoreaction Kinetics in Surface Freshwaters
Source: Molecules. 2019 Dec 18;25(1):9. doi: 10.3390/molecules25010009 (PMC7017383; doi:10.3390/molecules25010009)
Supplement: Supplementary file 1 [file molecules-25-00009-s001.zip › 3.2.4_gcc-4.4.0.zip!/3.2.4_gcc-4.4.0/3.2.4_gcc-4.4.0/doc/octave/html/interpreter/Introduction.html]

Introduction - Untitled


Next: Getting Started,
Previous: Preface,
Up: Top


---

## 1 A Brief Introduction to Octave

GNU Octave is a high-level language, primarily intended for numerical
computations. It provides a convenient interactive command line
interface for solving linear and nonlinear problems numerically, and
for performing other numerical experiments. It may also be used as a
batch-oriented language for data processing.

GNU Octave is freely redistributable software. You may redistribute
it and/or modify it under the terms of the GNU General Public License
as published by the Free Software Foundation. The GPL is included in
this manual in Copying.

This manual provides comprehensive documentation on how to install,
run, use, and extend GNU Octave. Additional chapters describe how
to report bugs and help contribute code.

This document corresponds to Octave version 3.2.4.

- Running Octave- Simple Examples- Conventions
